# Beclin1 and HMGB1 ameliorate the α-synuclein-mediated autophagy inhibition in PC12 cells

**DOI:** 10.1186/s13000-016-0459-5

**Published:** 2016-01-29

**Authors:** Kaihua Wang, Jianmin Huang, Wei Xie, Longjian Huang, Canhua Zhong, Zhenzhen Chen

**Affiliations:** Department of Neurology, RuiKang Hospital Affiliated to Guangxi University of Chinese Medicine, Nanning, Guangxi 530011 China; Southern Medical University, Guangzhou, Guangdong 510515 China; Department of Traditional Chinese Medicine, Nanfang Hospital, Southern Medical University, Guangzhou, Guangdong 510515 China; School of Traditional Chinese Medicine, Southern Medical University, Guangzhou, Guangdong 510515 China; Guangxi University of Chinese Medicine, Nanning, Guangxi 530001 China

**Keywords:** Beclin1, HMGB1, α-synuclein, Autophagy, Pheochromocytoma PC12 cells

## Abstract

**Background:**

Aberrant α-synuclein aggregation due to the deficiency of ubiquitin-proteasome or of autophagy characterizes the parkinson disease (PD). High mobility group box 1 (HMGB1) is a novel stress sensor to mediate the persistent neuro-inflammation and the consequent progressive neurodegeneration, via controlling the cellular autophagy/apoptosis checkpoint during inflammation. Moreover, HMGB1 has been recently indicated to involve in the autophagic degradation of α-synuclein.

**Methods:**

In the current study, we investigated the influence of the overexpressed α-synuclein of wild type (wt) or mutant type (A53T and A30P, mt) on the cytosolic levels of HMGB1 and Beclin1 and on the starvation-induced autophagy in pheochromocytoma PC12 cells. And then we explored the overexpression of HMGB1 or of Beclin1 on the α-synuclein degradation and on the autophagy in the α-synuclein-overexpressed PC12 cells.

**Results:**

It was demonstrated that α-synuclein overexpression inhibited the trans-location of HMGB1 from nucleus to cytosol and reduced the cytosolic level of Beclin1 in PC12 cells, and inhibited the starvation-induced autophagy via downregulating autophagy-associated markers and via reducing the autophagic vesicles in PC12 cells under starvation. On the other side, the intracellular promotion of either HMGB1 or Beclin1 upregulated the α-synuclein degradation and ameliorated the α-synuclein-mediated autophagy reduction in PC12 cells. However, the exogenous HMGB1 treatment exerted no such regulation in PC12 cells.

**Conclusion:**

In summary, our study confirmed the positive regulation by HMGB1 and Beclin1 on the α-synuclein degradation and on the starvation-induced autophagy in PC12 cells, implying both markers as prominent targets to promote the α-synuclein degradation.

## Background

Aberrant α-synuclein aggregation and the followed degeneration of dopaminergic cells [1–4] characterize the parkinson disease (PD). The deficient protein degradation pathways such as ubiquitin-proteasome system (UPS) and autophagy lysosomal pathway (ALP) have been recognized to contribute to the scanty degradation of wild-type (WT) or mutant-type (MT, A53T and A30P) α-synuclein [5, 6]. Moreover, autophagy is the only clearance pathway for the aggregated α-syn [7, 8]. And the both macromolecular complexes, mammalian target of rapamycin (mTOR)-Unc-51 like autophagy activating kinase 1 (ULK1) complex and the Beclin 1-Phosphatidylinositol 3-kinase catalytic subunit type 3 (PIK3C3) complex [9] are important for α-synuclein degradation [10].

Recently, multiple molecules have been characterized to regulate autophagy via the mTOR-ULK1 or PIK3C3 pathway. Amino acid starvation stimulates phosphorylation of ULK1 by protein phosphatase 2A to form PP2A-B55α complex and induces the ULK1-dependent autophagy [11, 12]. The transcriptional regulation of Annexin A2 also promotes the starvation-induced autophagy, via regulating autophagosome formation by enabling appropriate Autophagy-related protein (ATG) 9A trafficking from endosomes to autophagosomes [13]. Moreover, various markers have been confirmed to regulate autophagy through the PIK3C3 pathway. Dapper1 promotes autophagy by enhancing the Beclin1- vacuolar protein sorting 34 (VPS34)-Atg14L complex formation [14]. Interferon-alpha-2b induces autophagy in hepatocellular carcinoma cells via stimulating Beclin1 pathway [15]. Rho-associated, coiled-coil containing protein kinase 1 (ROCK1) promotes autophagy by binding and phosphorylating Beclin1 at Thr119 under metabolic stress [16]. However, Macrophage stimulating 1 (Mst1) inhibits autophagy by promoting the interaction between Beclin1 and Bcl-2 [17]. And Smad2 binds to the *Beclin1* promoter region, transcriptionally regulates autophagy via regulating beclin1 expression [18].

High mobility group box 1 (HMGB1) is a stress sensor that plays a critical role in various physiological and pathological processes including cell development, differentiation, inflammation, metabolism and death [19]. Increasing evidence demonstrates that HMGB1-dependent autophagy promotes chemotherapy resistance [20], sustains tumor metabolism [21], protects against endotoxemia [22] and involves in other pathological processes [23, 24]. Inflammation has also been recognized to involve in PD [25–27]. Sustained inflammatory process and the activated microglia might drive the progressive degeneration of dopamine neurons in PD [28]. Moreover, the cytoplasmic HMGB1 has been indicated to control the cellular autophagy/apoptosis checkpoint during inflammation [29]. HMGB1 was demonstrated to mediate the persistent neuroinflammation and consequent progressive neurodegeneration, via stimulating the production of multiple inflammatory and neurotoxic factors [30]. In addition, HMGB1 has been recently indicated to involve in the autophagy inhibition caused by α-synuclein overexpression [31], implying a direct role in modulating autophagic degradation of α-synuclein.

In current study, we investigated the regulation of α-synuclein overexpression on the trans-location or the expression of HMGB1 and Beclin1, and on the starvation-induced autophagy in pheochromocytoma PC12 cells. And then we explored the overexpression of HMGB1 or Beclin1 on the α-synuclein degradation and on the autophagy in the α-synuclein-overexpressed PC12 cells. This study confirmed the positive regulation by endogenous HMGB1 on the autophagy-mediated α-synuclein degradation in PC12 cells.

## Methods

### Construction of PC12 (Syn^wt^) and PC12 (Syn^mt^) cells

Rattus norvegicus pheochromocytoma PC12 cell line was purchased from American Type Culture Collection (ATCC) (Rockville, MD, USA) and was cultured in RPMI-1640 medium (GIBCO, Rockville, MD, USA) supplemented with 10 % (2 % for maintaining) fetal bovine serum (FBS) (Hyclone, Pittsburgh, PA, USA) and with 100 U/mL penicillin and 100 mg/mL streptomycin (CSPC Pharmaceutical Group Limited, China). Cells were cultured at 37 °C in a humidified incubator with 5 % CO_2_. To construct an α-synuclein-overexpressed PC12 cell line, the wild-type (wt) (NM_019169.2), mutant-type (mt, A53T and A30P) α-synuclein coding sequence, or the Enhanced Green Fluorescence Protein (EGFP) coding sequence (as control) was amplified and cloned into the pcDNA3.1(+) vector (Invitrogen, Carlsbad, CA, USA). Then we transfected the α-Syn(wt)-pcDNA3.1(+), α-Syn(mt)-pcDNA3.1(+) or EGFP-pcDNA3.1(+) plasmid into the 85 % confluent PC12 cells with lipofectamine 2000 (Invitrogen, Carlsbad, CA, USA). After the transfection for 24 hours, cells were updated with the RPMI-1640 medium which were supplemented with 10 % FBS and with 1.2 mg/ml G418 (Life Technologies, Grand Island, NY, USA) to select the cell clone overexpressing wt or mt α-synuclein or EGFP. And the selected PC12 (Syn^wt^), PC12 (Syn^mt^) and PC12 (Con) cell clones were cultured in RPMI-1640 medium + 10 % FBS supplemented with 0.8 mg/ml G418. For the starvation treatment, PC12 (Syn^wt^), PC12 (Syn^mt^) and PC12 (Con) cells were inoculated with FBS-free RPMI-1640 medium at 37 °C in a humidified chamber with 5 % CO_2_.

### Overexpression of HMGB1 or Beclin 1 in PC12 (Syn^wt^) or PC12 (Syn^mt^) cells

To promote the HMGB1 level in PC12 (Syn^wt^) or PC12 (Syn^mt^) cells, the coding sequence of HMGB1 or the coding sequence of Red Fluorescence Protein (RFP) was cloned into the pcDNA3.1(+) vector. And 5 μg/mL (to guarantee more than 90 % cells to be transfected) HMGB1-pcDNA3.1(+) or RFP-pcDNA3.1(+) plasmid was transfected into 10^5^ per well 85 % confluent PC12 (Syn^wt^) or PC12 (Syn^mt^) cells. To upregulate the Beclin1 level in PC12 cells, Beclin1 or the coding sequence of Chloramphenicol acetyl transferase(CAT) was amplified and was cloned into the pLenti 6/TR vector (Invitrogen, Carlsbad, CA, USA). Recombinant pLenti-Beclin1 (LV-Beclin1) or pLenti-CAT virus of (LV-Con) was produced by cotransfecting 293 T cells with pLenti-Beclin1 or pLenti-CAT and ViraSafe™ Lentiviral Packaging System (Cell Biolabs, San Diego, CA, USA). PC12 (Syn^wt^) or PC12 (Syn^mt^) cells were infected with LV-Beclin1 or LV-Con virus with 1 Multiplicity of infection (MOI) for 12 or 24 hours.

### mRNA isolation and real-time PCR analysis of α-synuclein mRNA

mRNA samples from OVCAR-3 cells were prepared with Kit for mRNA Isolation and Purification (Clontech, Palo Alto, CA, USA), and reverse transcription (RT) was performed with M-MLV Reverse Transcriptase (Promega, Madison, WI, USA) as following: 42 °C for 5 min and 95 °C for 10 sec for the reverse transcription. And the real-time PCR assay was performed at 95 °C for 5 sec and 60 °C for 20 sec for the PCR reaction, with 40 cycles. The primer sequences for α-synuclein were as following: forward primer, 5’- CGT CCT CTA TGT AGG TTC CA -3’, reverse primer: 5’- GCC ACT GTT GTC ACT CCA TG -3’. The primer sequences for β-actin were as following: forward primer, 5’- GTA CCC TGG CAT TGC CGA CA -3’, reverse primer: 5’- GGA CTC GTC ATA CTC CTG CTT GCT -3’. Relative α-synuclein mRNA level was normalized to internal control β-actin, with the ∆∆Ct method [32].

### Western blotting assay

PC12, PC12 (Con), PC12 (Syn^wt^) or PC12 (Syn^mt^) cells were homogenized with an ice-cold NE-PER Nuclear and Cytoplasmic Extraction Reagents Kit (Pierce, Rockford, IL, USA), the nuclear and the cytoplasmic proteins were isolated according to the product’s manual. Each protein sample was supplemented with a protease Inhibitor Cocktail (Abcam, Cambridge, UK) and was stored at -80 °C before use. The western blotting assay was performed with the rabbit polyclone antibody against α-synuclein (Abcam, Cambridge, UK), against HMGB1(Abcam, Cambridge, UK), against Beclin1 (Cell Signaling Technology Inc., Danvers, MA, USA), against LC3 (Pierce, Rockford, IL, USA), Atg 7 (Sigma-Aldrich, St. Louis, MO, USA), against mTOR (Abcam, Cambridge, UK), against LMNB1 (Pierce, Rockford, IL, USA) or against β-actin (Sinobio, Beijing, China), and with horseradish peroxidase-linked secondary antibodies (Jackson ImmunoResearch, West Grove, PA, USA). The specific binding band was scanned and quantified according to the band density.

### Quantitative EGFP-LC3 analysis and electron microscopy

1 × 10^5^ cells/well PC12, PC12 (Con), PC12 (Syn^wt^) or PC12 (Syn^mt^) cells were transfected with 2 μg EGFP-LC3 reporter plasmid, pcDNA3.1-EGFP-LC3, with Lipofectamine 2000 (Invitrogen, Carlsbad, CA, USA) for a 24 hour’s incubation to quantify the EGFP-positive autophagic vesicles which were promoted by various treatments. Then the EGFP-positive vesicles were visualized under fluorescence microscopy (JEM1230, Japan).

### Statistical evaluations

Quantitative data was presented as mean ± SE and was analyzed for a significant difference with the Student’s *t* test or one way ANOVA test. A *p* value less than 0.05 was considered to be statistically significant.

## Results

### α-synuclein overexpression inhibits the trans-location of HMGB1 and the expression of Beclin1 in PC12 cells

**Fig. 1:**
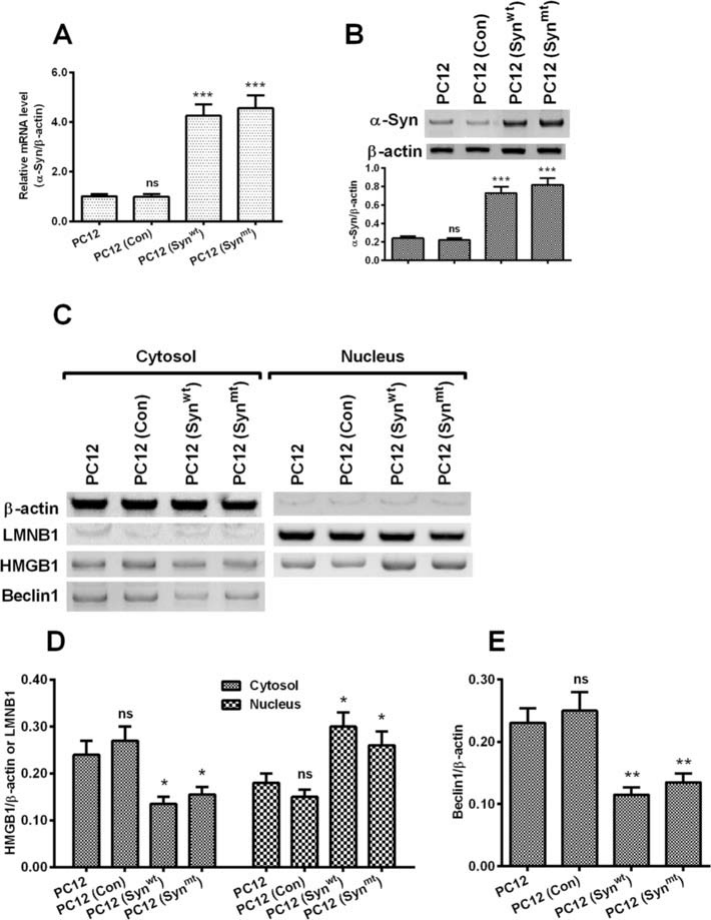
Overexpression of α-synuclein with wild type (wt) or mutant type (mt) reduces cytosolic HMGB1 and Beclin1 in PC12 cells. **a** and **b**: mRNA (**a**) and protein (**b**) levels of α-synuclein in the PC12 cell overexpressing wt α-synuclein, PC12 (Syn^wt^), in the PC12 cell overexpressing mt (A53T and A30P) α-synuclein, PC12 (Syn^mt^), in the PC12 cell overexpressing Enhanced Green Fluorescence Protein (EGFP), PC12 (Con), or in the blank P12 cells, the protein level of α-synuclein was examined by western blot analysis; **c**: Western blot analysis of HMGB1 (in cytosol and in nucleus) and Beclin1 (in cytosol) in PC12, PC12 (Con), PC12 (Syn^wt^) or PC12 (Syn^mt^) cells; **d**: Relative HMGB1 level to β-actin in cytosol or to Lamin B(LMNB1) in the nucleus of PC12, PC12 (Con), PC12 (Syn^wt^) or PC12 (Syn^mt^) cells; **e**: Relative Beclin1 level to β-actin in the cytosol of PC12, PC12 (Con), PC12 (Syn^wt^) or PC12 (Syn^mt^) cells; Each result was averaged for triple independent experiments. Statistical significance was presented as * *p* < 0.05, ** *p* < 0.01, *** *p* < 0.001, ns: no significance

To investigate the regulation of α-synuclein overexpression on the trans-location or the level of HMGB1 and Beclin1, and on the starvation-induced autophagy in pheochromocytoma PC12 cells, we constructed the PC12 cell overexpressing WT α-synuclein, PC12 (Syn^wt^), or the PC12 cell overexpressing MT (A53T and A30P) α-synuclein, PC12 (Syn^mt^), with PC12 (Con), which overexpressed enhanced green fluorescent protein (EGFP), as control. As shown in Fig. 1a, the mRNA level of α-synuclein was significantly higher in both PC12 (Syn^wt^) and PC12 (Syn^mt^) cells, compared with the PC12 (Con) or blank PC12 cells (either *p* < 0.001 for PC12 (Syn^wt^) or PC12 (Syn^mt^) cells). And the protein level of α-synuclein was confirmed by the western blot analysis in the PC12 (Syn^wt^) or PC12 (Syn^mt^) cells (*p* < 0.001 respectively, Fig. 1b). And then we investigated the influence of α-synuclein overexpression on the Beclin1 level and the trans-location of HMGB1 from nucleus to cytosol. Western blotting (Fig. 1c) demonstrated that the cytosolic HMGB1 was markedly upregulated, whereas was significantly downregulated in nucleus in either PC12 (Syn^wt^) or PC12 (Syn^mt^) cells (*p* < 0.05 respectively, Fig. 1c). In addition the cytoplasmic Beclin1 was also examined with western blot analysis. It was indicated that the Beclin level was significantly downregulated in both PC12 (Syn^wt^) and PC12 (Syn^mt^) cells (*p* < 0.01 respectively, Fig. 1d). Thus, we confirmed the promotion to the trans-location of HMGB1 from nucleus to cytosol, and to the cytoplasmic level of Beclin1 in PC12 cells.

### α-synuclein overexpression inhibits autophagy in PC12 cells

**Fig. 2:**
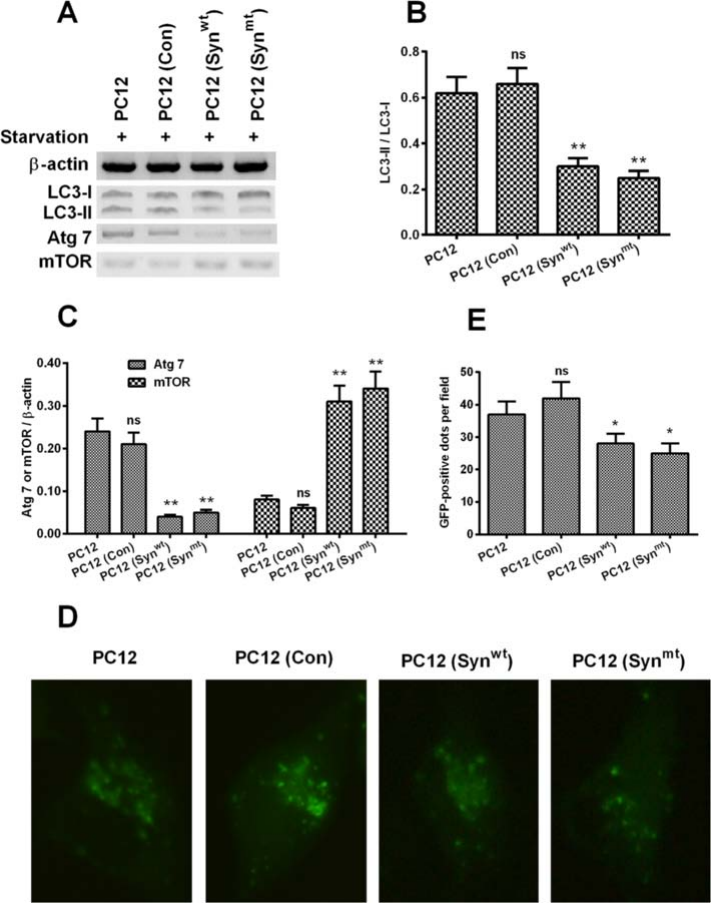
Levels of autophagy-associated markers and autophagic vesicles induced by starvation in the PC12, PC12 (Con), PC12 (Syn^wt^) or PC12 (Syn^mt^) cells. **a**: Western blotting assay of LC3-I, LC3-II, Atg 7 and mTOR in the PC12, PC12 (Con), PC12 (Syn^wt^) or PC12 (Syn^mt^) cells post an incubation at 37 °C in FBS-free medium for 24 hours; **b**: Ratio of LC3-II to LC3-I in the starvation-treated cell lines; **c**: Relative level of Atg 7 and mTOR levels to β-actin in each cell line post the starvation treatment; **d** and **e**: Imaging (**d**) and counting (**e**) of EGFP-positive autophagic vesicles in the cytosol of starvation-treated PC12, PC12 (Con), PC12 (Syn^wt^) or PC12 (Syn^mt^) cells which were transfected with pCDNA3.1-EGFP-LC3 plasmid for another 24 hours; Data was averaged for triple independent results. * *p* < 0.05, ** *p* < 0.01, ns: no significance

We then examined the regulation of the overexpressed α-synuclein on the autophagy in PC12 cells. The autophagy induction in PC12, PC12 (Con), PC12 (Syn^wt^) or PC12 (Syn^mt^) cells subject to starvation was examined with western blotting assay and the EGFP-LC3 reporter assay. Western blotting results (Fig. 2a) demonstrated that the conversion of LC3-I to LC3-II was markedly lower in either PC12 (Syn^wt^) or PC12 (Syn^mt^) cells, compared to the normal PC12 or the PC12 (Con) cells (*p* < 0.01 respectively, Fig. 2b). And such reduced autophagy was also confirmed by the reduced level of Atg7 and increased level of mTOR in either PC12 (Syn^wt^) or PC12 (Syn^mt^) cells (*p* < 0.01 respectively, Fig. 2b). Moreover, the EGFP-LC3 reporter assay also demonstrated that there were less GFP-positive autophagic puncta in either PC12 cells. Taken together, the α-synuclein overexpression with wild or mutant type inhibits the starvation-stimulated autophagy in PC12 cells.

### HMGB1 promotes autophagy and α-synuclein degradation in the α-synuclein-overexpressed PC12 cells

**Fig. 3:**
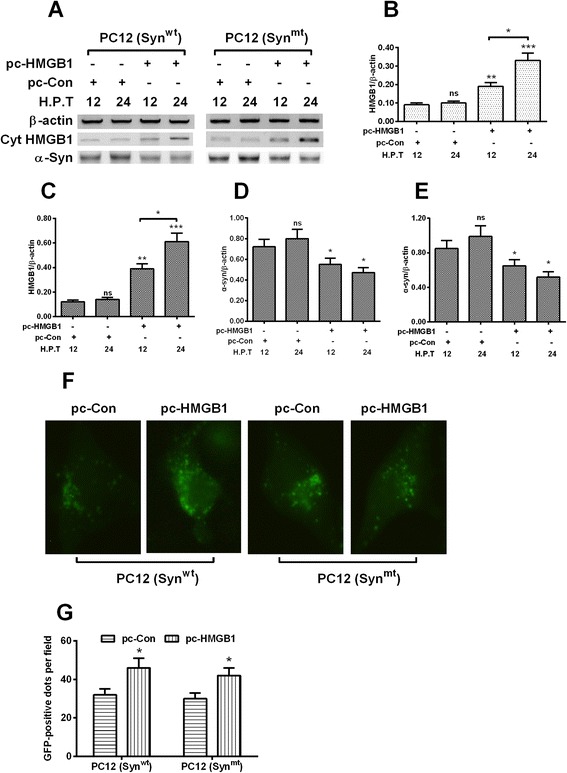
HMGB1 upregulation inhibits α-synuclein accumulation and ameliorates the α-synuclein-inhibited autophagy in PC12 (Syn^wt^) and PC12 (Syn^mt^) cells. **a**: Western blot analysis of HMGB1 and α-synuclein in PC12 (Syn^wt^) and PC12 (Syn^mt^) cells, which were transfected with HMGB1-pcDNA3.1(+) or RFP-pcDNA3.1(+) plasmid for 12 or 24 hours; **b** and **c**: Ratio of HMGB1 to β-actin in PC12 (Syn^wt^) and PC12 (Syn^mt^) cells with or without HMGB1 promoted; **d** and **e**: Ratio of α-synuclein to β-actin in PC12 (Syn^wt^) and PC12 (Syn^mt^) cells, with or without HMGB1 promoted; **f** and **g**: Imaging (**f**) and counting (**g**) of EGFP-positive autophagic vesicles in the cytosol of starvation-treated PC12 (Syn^wt^) or PC12 (Syn^mt^), with or without HMGB1 promoted. Each result was averaged for triple independent experiments. Statistical significance was presented as * *p* < 0.05, ** *p* < 0.01, *** *p* < 0.001, ns: no significance

To investigate the role of HMGB1 on the autophagic degradation of α-synuclein, we overepressed HMGB1 in both PC12 (Syn^wt^) and PC12 (Syn^mt^) cells with the gain-of-function strategy, and then re-evaluated the starvation-induced autophagy in both cell lines. Western blotting assay (Fig. 3a) demonstrated that the transfection with HMGB1-pcDNA3.1(+) (pc-HMGB1) dramatically upregulated the HMGB1 level in both PC12 (Syn^wt^) (Fig. 3b) and PC12 (Syn^mt^) (Fig. 3c) cells at 12 or 24 hour post transfection (H.P.T.) (*p* < 0.01 or *p* < 0.001), and time-dependently (*p* < 0.05). However, the α-Synuclain level was markedly downregulated in both PC12 (Syn^wt^) (Fig. 3d) and PC12 (Syn^mt^) (Fig. 3e) cells (*p* < 0.05 respectively). Moreover, the EGFP-LC3 reporter assay (Fig. 3f) demonstrated that the overexpressed HMGB1 promoted more autophagic vesicles in either PC12 (Syn^wt^) or PC12 (Syn^mt^) cells which were subject to starvation. Therefore, HMGB1 promotes α-synuclein degradation and ameliorates the α-synuclein-mediated autophagy reduction in PC12 cells.

**Fig. 4:**
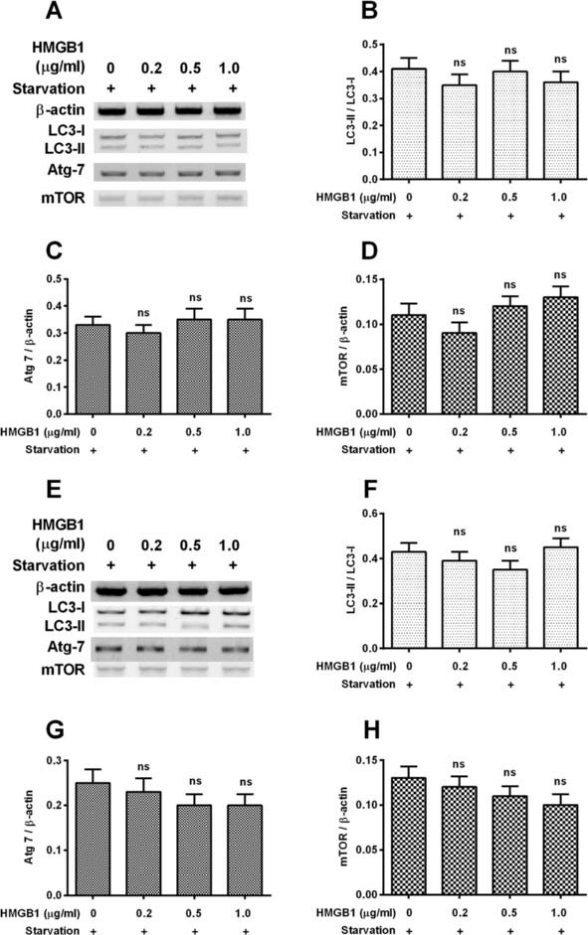
Western blot analysis of autophagy-associated markers in the PC12 (Syn^wt^) or the PC12 (Syn^mt^) cells, which were treated with HMGB1. **a**: Western blotting assay of LC3-I, LC3-II, Atg 7 and mTOR in the PC12 (Syn^wt^) cells which were treated with 0, 0.2, 0.5 or 1 μg/mL HMGB1, under starvation for 24 hours; **b**: Ratio of LC3-II to LC3-I in the starvation-treated PC12 (Syn^wt^) cells with HMGB1 treatment; **c** and **d**: Relative level of Atg 7 (**c**) and mTOR (**d**) levels to β-actin in the PC12 (Syn^wt^) cells with HMGB1 treatment; **e**: Western blotting assay of LC3-I, LC3-II, Atg 7 and mTOR in the PC12 (Syn^mt^) cells which were treated with 0, 0.2, 0.5 or 1 μg/mL HMGB1, under starvation for 24 hours; **f**: Ratio of LC3-II to LC3-I in the starvation-treated PC12 (Syn^mt^) cells with HMGB1 treatment; **g** and **h**: Relative level of Atg 7 (**g**) and mTOR (**h**) levels to β-actin in the PC12 (Syn^mt^) cells with HMGB1 treatment. Data was averaged for triple independent results, ns: no significance

In addition, we also investigated the influence of exogenous HMGB1 treatment on the starvation-induced autophagy in PC12 cells. Western blotting assay (Fig. 4a) demonstrated that the treatment with 0.2 to 1 μg/mL HMGB1 did not significantly regulate the conversion of LC3-I to LC3-II (Fig. 4b) and the expression of Atg 7 (Fig. 4c) and mTOR (Fig. 4d). Therefore, the exogenous HMGB1 exerts no regulation on the starvation-induced autophagy in PC12 cells.

### Beclin1 overexpression promotes autophagy and α-synuclein degradation in the α-synuclein-overexpressed PC12 cells

**Fig. 5:**
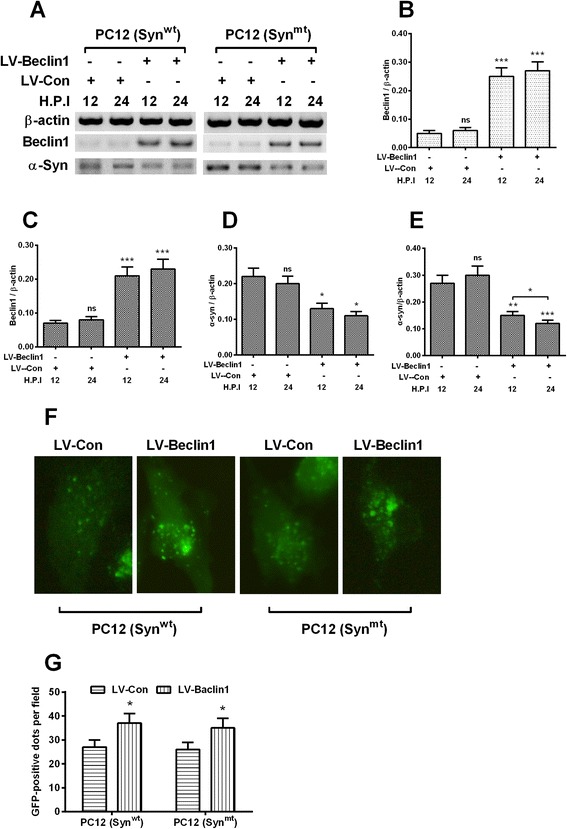
Beclin1 upregulation reduces α-synuclein accumulation and ameliorates the α-synuclein-inhibited autophagy in PC12 (Syn^wt^) and PC12 (Syn^mt^) cells. **a**: Western blot analysis of Beclin1 and α-synuclein in the PC12 (Syn^wt^) and PC12 (Syn^mt^) cells which were infected with 1 multiplicity of infection (MOI) pLenti-Beclin1 (LV-Beclin1) or pLenti-Con (LV-Con) under starvation for 12 or 24 hours; **b** and **c**: Ratio of Beclin1 to β-actin in PC12 (Syn^wt^) (**b**) and PC12 (Syn^mt^) (**c**) cells which were infected with LV-Beclin1 or with LV-Con virus; **d** and **e**: Ratio of α-synuclein to β-actin in PC12 (Syn^wt^) (**b**) and PC12 (Syn^mt^) (**c**) cells which were infected with LV-Beclin1 or with LV-Con virus; **f** and **g**: Imaging (**f**) and counting (**g**) of EGFP-positive autophagic vesicles in the starvation-treated PC12 (Syn^wt^) or PC12 (Syn^mt^), which were infected with LV-Beclin1 or with LV-Con virus. Each result was averaged for triple independent experiments. Statistical significance was presented as * *p* < 0.05, ** *p* < 0.01, *** *p* < 0.001, ns: no significance

Given the high importance of Beclin1-dependent autophagy in the α-synuclein degradation [10, 12], we then investigated the influence of Beclin1 overexpression on the α-synuclein degradation and the starvation-induced autophagy in both PC12 (Syn^wt^) and PC12 (Syn^mt^) cells. As shown in Fig. 5a, the lentivirus-mediated Beclin1 overexpression in protein level was significant in both cell lines (Fig. 5b and c) at either 12 or 24 H.P.I. (*p* < 0.001 respectively) with 1 multiplicity of infection (MOI) with the Beclin1-lentivirus (LV-Beclin1). And the α-synuclein level was markedly reduced at 12 or 24 H.P.I. in the LV-Beclin1-infected PC12 (Syn^wt^) (either *p* < 0.05 for 12 or 24 H.P.I., Fig. 5d) or PC12 (Syn^mt^) cells (*p* < 0.01 or *p* < 0.001 for 12 or 24 H.P.I., Fig. 5e). In addition, we examined the starvation-induced autophagic vesicles in each cell line with the EGFP-LC3 reporter assay. It was indicated in Fig. 5f and g that the infection with 1 MOI LV-Beclin1 promoted more autophagic vesicles than the LV-Con infection in either PC12 (Syn^wt^) or PC12 (Syn^mt^) cells (*p* < 0.05). Thus, we also confirmed the positive regulation by Beclin1 on the starvation-induced autophagy in PC12 cells.

## Discussion

HMGB1, as a cytokine-like factor [33], is increasingly recognized as a novel autophagy regulator via interfering with the PIK3C3 complex [34]. Nucleus-to-cytosol translocated HMGB1 competitively binds to Beclin1 and subsequently induces autophagy [35]. However, α-synuclein has been shown to impair the autophagy [36, 37]. In particular, α-synuclein could bind to both cytoplasmic and nuclear HMGB1, block HMGB1-Beclin1 binding, whereas to strengthen the Beclin1**-**BcL2 binding [31], and thus to inhibit autophagy. And such inhibition could be restored by the Beclin 1 overexpression. In the present study, we found that the overexpression of either WT or MT α-synuclein markedly reduced the cytoplasmic levels of both HMGB1 and Beclin1 in PC12 cells, implying the reduced HMGB1 and Beclin1 might contribute to the α-synuclein-mediated autophagy inhibition in PC12 cells.

Impaired autophagy has been indicated to correlate with the α-synuclein aggregation and neurodegeneration in PD [38–41]; and the stimulated autophagy could reduce the accumulation of α-synuclein in cells and in mouse brain [12, 42], and could even rescue midbrain dopamine neurons from α-synuclein toxicity [43]. On the other side, α-synuclein has been shown to impair autophagy [36, 37]. The overexpression of either WT or MT α-synuclein inhibits autophagy in pheochromocytoma PC12 cells [31], impairs neurite outgrowth of primary midbrain neurons, affects neurite branching [44]. In this study, we reconfirmed such autophagy inhibition by α-synuclein overexpression in PC12 cells, the overexpression of wild-type or mutant-type α-synuclein significantly downregulated the starvation-induced autophagy via inhibiting the mTOR/Atg 7 signaling. Therefore, we also confirmed the autophagy inhibition by the overexpressed α-synuclein in PC12 cells. And it implies that the reduced HMGB1 and Beclin1 might contribute to the reduced autopahgy in the α-synuclein-overexpressed PC12 cells.

HMGB1 is also known as the high-mobility group protein 1 (HMG-1). As a chromatin-associated nuclear protein, it is a critical regulator of autophagy. And the pharmacological inhibition of HMGB1 cytoplasmic translocation limits starvation-induced autophagy. Moreover, only endogenous HMGB1 has been indicated to regulate the Bcl-2-Beclin1 binding, via the direct interact with Beclin 1 [34, 35], and thus regulating the convergence of autophagy and apoptosis, via interacting with anti-apoptotic Bcl-2-like proteins [45]. Our study confirmed that either endogenous HMGB1 or Beclin 1 overexpression promoted the α-synuclein degradation in PC12 cells. Moreover, the overexpressed HMGB1 or Beclin 1 markedly ameliorated the α-synuclein-mediated autophagy reduction in the α-synuclein-overexpressed (either WT or MT) PC12 cells, implying the promotion by HMGB1 to the α-synuclein degradation might be autophagy-dependent. And such amelioration might be dependent on the HMGB1-Beclin1 interaction.

Interesting, our results indicated that the extracellular HMGB1 exerted no regulatory role on the starvation-induced autophagy, posing no influence on the levels of mTOR and Atg 7 in both PC12 (Syn^wt^) and PC12 (Syn^mt^) cells. HMGB1 is a secretary cytokine from activated macrophages and monocytes [46] to mediate inflammation [33], via binding to receptor for advanced glycation endproducts (RAGE) [47] or to toll-like receptor (TLR) [48]. However, the present study confirmed that the promotion by HMGB1 was not RAGE- or TLR-dependent. In addition, the gain-of-function strategy also confirmed the promotion to the α-synuclein degradation and the autophagy induction by Beclin1 overexpression in PC12 cells. Taken together, we speculated that endogenous HMGB1 and Beclin1 might promote the autophagic degradation of α-synuclein. Therefore, the endogenous HMGB1 and Beclin1 exerts protective role in the cells against the α-synuclein accumulation.

## Conclusion

In summary, α-synuclein with wild-type or mutant-type inhibited autophagy in PC12 cells via inhibiting the HMGB1 and Beclin1. On the other side, the cytosolic promotion to HMGB1 or to Beclin1 up-regulates the autophagic degradation of α-synuclein via increasing the autophagy in PC12 cells. Therefore, the endogenous HMGB1 and Beclin1 present protective role in the cells against the α-synuclein accumulation.

## Abbreviations

PD: Parkinson disease
HMGB1: High mobility group box 1
ALP: Autophagy lysosomal pathway
WT: Wild-type
(MT: A53T and A30P), Mutant-type
ULK1: Unc-51 like autophagy activating kinase 1
mTOR: Mammalian target of rapamycin
PIK3C3: Beclin 1-Phosphatidylinositol 3-kinase catalytic subunit type 3
ATG: Autophagy-related protein
VPS34: Vacuolar protein sorting 34
ROCK1: Rho-associated, coiled-coil containing protein kinase 1
Mst1: Macrophage stimulating 1
PC12 (Syn^mt^): PC12 cell overexpressing MT (A53T and A30P) α-synuclein
PC12 (Syn^wt^): PC12 cell overexpressing WT α-synuclein
